# Characterization of immortalized MARCO and SR-AI/II-deficient murine alveolar macrophage cell lines

**DOI:** 10.1186/1743-8977-5-7

**Published:** 2008-05-02

**Authors:** Hongwei Zhou, Amy Imrich, Lester Kobzik

**Affiliations:** 1 Department of Environmental Health, Harvard School of Public Health, Boston, MA, 02115, USA; 2 Department of Pathology, Brigham and Women's Hospital, Harvard Medical School, Boston, MA 02115, USA

## Abstract

**Background:**

Alveolar macrophages (AM) avidly bind and ingest unopsonized inhaled particles and bacteria through class A scavenger receptors (SRAs) MARCO and SR-AI/II. Studies to characterize the function of these SRAs have used AMs from MARCO or SR-AI/II null mice, but this approach is limited by the relatively low yield of AMs. Moreover, studies using both MARCO and SR-AI/II-deficient (MS^-/-^) mice have not been reported yet. Hence, we sought to develop continuous cell lines from primary alveolar macrophages from MS^-/- ^mice.

**Results:**

We used *in vitro *infection of the primary AMs with the J2 retrovirus carrying the *v-raf *and *v-myc *oncogenes. Following initial isolation in media supplemented with murine macrophage colony-stimulating factor (M-CSF), we subcloned three AM cell lines, designated ZK-1, ZK-2 and ZK-6. These cell lines grow well in RPMI-1640-10% FBS in the absence of M-CSF. These adherent but trypsin-sensitive cell lines have a doubling time of approximately 14 hours, exhibit typical macrophage morphology, and express macrophage-associated cell surface Mac-1 (CD11b) and F4/80 antigens. The cell lines show robust Fc-receptor dependent phagocytosis of opsonized red blood cells. Similar to freshly isolated AMs from MS^-/- ^mice, the cell lines exhibit decreased phagocytosis of unopsonized titanium dioxide (TiO_2_), fluorescent latex beads and bacteria (*Staphylococcus aureus*) compared with the primary AMs from wild type (WT) C57BL/6 mice.

**Conclusion:**

Our results indicated that three contiguous murine alveolar macrophage cell lines with MS^-/- ^(ZK1, ZK2 and ZK6) were established successfully. These cell lines demonstrated macrophage morphology and functional activity. Interestingly, similar to freshly isolated AMs from MS^-/- ^mice, the cell lines have a reduced, but not absent, ability to bind and ingest particles, with an altered pattern of blockade by scavenger receptor inhibitors. These cell lines will facilitate *in vitro *studies to further define MARCO and SR-AI/II function, and may also be useful to identify other novel scavenger-type macrophage receptors and for additional studies of particle toxicology.

## Background

The pulmonary alveolar macrophage (AM) plays an important role in defense of the lung [1-5]. Class A scavenger receptors (SRA) primarily expressed on the macrophage (MØ) surface are critical for binding, uptake, and response to inhaled unopsonized environmental particles (*e.g*. TiO2) and microbes [6-11]. The SRA defines a group of pattern recognition receptors composed of three members as follows: SR-AI/II [12], macrophage receptor with collagenous structure (MARCO) [13], and scavenger receptor with C-type lectin (SRCL) [14]. All are multifunctional trimeric glycoproteins, and they are able to bind and internalize a broad range of ligands such as Gram-negative bacteria (lipopolysaccharide), Gram-positive bacteria (lipoteichoic acid) and modified lipoproteins *etc *[15-18]. Investigation of the function of these SRAs has used AMs from MARCO or SR-AI/II-deficient mice [9,19], but this approach has been impeded by the relatively low yield of AMs recoverable from animals by laborious procedures, and by the heterogeneity of freshly isolated macrophage. To overcome such obstacles, the *in vitro *establishment of cell lines maintaining differentiated functions has provided a very important tool to facilitate biological study of macrophages [20-23].

Several murine macrophage cell lines from bone marrow [24,25], spleen [26,27], fetal liver [28,29], and lung [30] have been successfully obtained by *in vitro *infection of primary cell cultures with a recombinant J2 retrovirus carrying the *v-raf *and *v-myc *oncogenes. In addition, investigation of the function of both MARCO and SR-AI/II using MS^-/- ^mice has not been reported yet. These observations prompted us to develop a continuous alveolar macrophage cell line with MARCO and SR-AI/II deficient using the J2 retrovirus. This report describes the establishment, growth characteristics, morphological and functional characterization of a continuous line of alveolar macrophages which was derived from brochoalveolar lavage (BAL) obtained from MS^-/- ^mice [31]. Immortalization was conducted by infection of the primary AMs from MS^-/- ^mice with a retrovirus J2. The immortalized AMs were cloned by limiting dilution method. Three of the clones, designated as ZK-1, ZK-2 and ZK-6 were chosen for further characterization of macrophage phenotype and phagocytic function.

## Results

### ZK cells are SR-AI/II and MARCO-deficient

**Figure 1:**
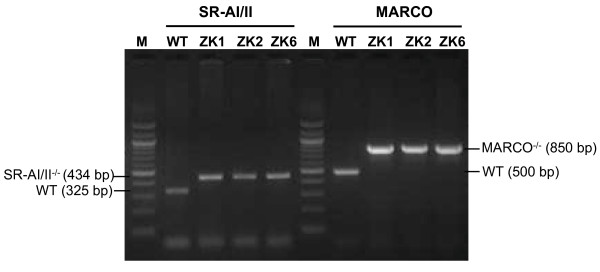
ZK1, ZK2 and ZK6 cell lines are MARCO^-/- ^and SR-AI/II^-/- ^(MS^-/-^) by PCR genotyping. With primers for SR-A, amplifies a 325 bp DNA fragment from the C57BL/6 wild-type (WT) allele; with SR-AI/II mutant allele primers, amplifies a 434 bp DNA fragment from SRA-deficient ZK1, ZK2 and ZK6 cells. With primers for MARCO wild-type allele, amplifies a 500 bp DNA fragment from WT mice; with primers for MARCO mutant allele, amplifies a 850 bp DNA fragment from ZK cells. ZK1, ZK2 and ZK6 clones exhibited both MARCO and SRA-I/II-deficient. PCR products, ca.10 μl/each was resolved on a 1.5% agarose gel by gel electrophoresis. M, 100 bp DNA marker.

The three clones designated as ZK1, ZK2 and ZK6 were obtained by limiting dilution and examined for their growth characteristics, surface phenotype, and functional properties. PCR genotyping of these cell lines confirmed that they are SR-AI/II^-/- ^and MARCO^-/- ^(Fig. 1). SR-AI/II wild-type allele exhibited a 325-bp PCR product, whereas SR-AI/II^-/- ^mutant allele showed a 434-bp PCR product. MARCO wild type allele exhibited ca. 500-bp PCR product, and MARCO^-/- ^mutant exhibited ca. 850-bp PCR product. All of the three cell lines are stable and Mycoplasma-free by Mycoplasma PCR ELISA test (Roche, Indianapolis, IN) during culture in the past 24 months.

### Growth rate of ZK cells

**Figure 2:**
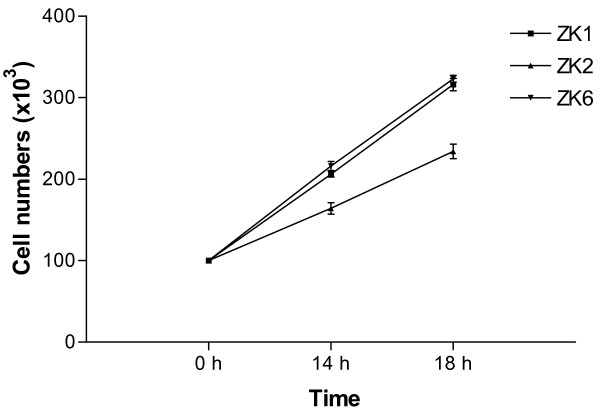
Growth rate of the continuous murine alveolar macrophage single cell clones ZK1 (◆), ZK2 (■) and ZK6 (▲). Cells were seeded in six-well plate at 2 × 10^4 ^cells/ml and incubated at 37°C in a 5% CO_2_-humidified atmosphere in RPMI/10% FBS complete medium. Three wells per clone were harvested with cold PBS at 14 h and 18 h post seeding, and cells from each well were counted by a hemocytometer with trypan blue exclusion of dead cells. The obtained average cell count for each clone at each time point was plotted against the time. Doubling time (mean generation time = mgt) was calculated according the formula: N = N_0_2^T/mgt^. N is the number of cells at any time T; N_0_, is the number of cells at an initial point. The doubling time of ZK cell lines is approximately 14 hours.

All three ZK cell lines replicate rapidly in regular RPMI medium or DMEM with 10% FBS in the absence of a specific growth factor supplement. Doubling time (mean generation time = mgt) was calculated according the formula: N = N_0_2^T/mgt^. On the average, doubling time of ZK cell lines is between 12 h to 16 h in RPMI complete medium (Fig. 2). Like their parental primary AMs isolated from the MS^-/- ^mice, all of the cell lines are adherent but trypsin-sensitive for passage.

### Morphology

**Figure 3:**
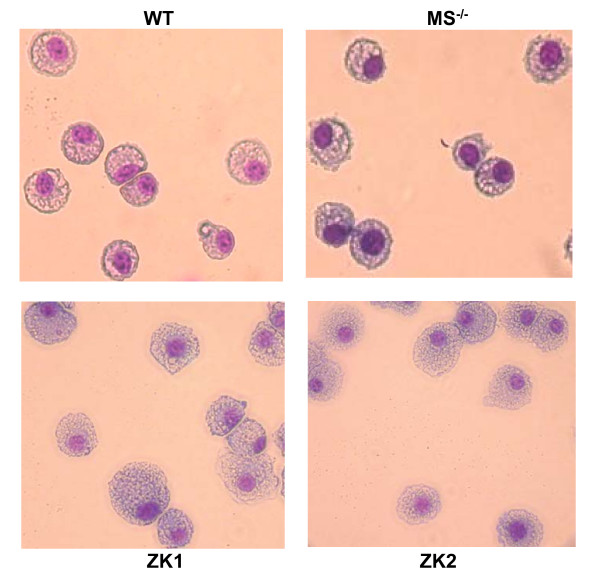
Modified Wright staining of ZK1 and ZK2 cell lines compared with primary alveolar macrophages. Primary AMs were isolated from wild type (WT) C57BL/6 mice and from MARCO^-/- ^and SR-AI/II^-/- ^(MS^-/-^) mice. ZK1 and ZK2 cells were identified as macrophages by their large, dark nuclei and abundant pale, granular cytoplasm containing numerous vacuoles.

Light microscopic examination of Diff Quik, a modified Wright-stained cytospin slides of ZK cells showed cells of considerable homogeneity in size and shape. All ZK cells had large, dark, round or oval nuclei with abundant pale cytoplasm containing small granules and numerous cytoplasmic vacuoles (Fig. 3). Only few cells showed double nuclei that may indicate cell division. Primary AMs isolated from wild-type mice and MS^-/- ^mice by BAL contained at least 95% macrophages, and ZK cells demonstrated the same morphology as these primary AMs.

### Expression of macrophage-associated cell surface antigens

**Figure 4:**
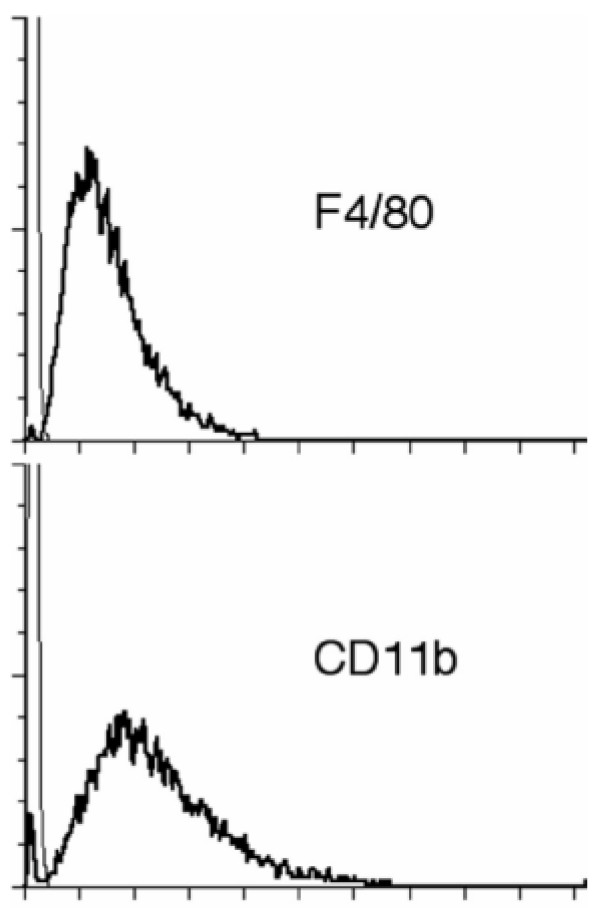
Expression of F4/80 and Mac-1 (CD11b) antigens by ZK1 cells, as determined by flow-cytometry. Cell surface expression of the macrophage-associated differentiation Ags was assessed by direct immunofluorescence (thick lines). ZK1 cells were incubated with FITC-labeled anti-mouse F4/80 or FITC-labeled anti-mouse CD11b. Mouse IgG2a and IgG2b were used as isotype controls (light lines). Staining of cells with FITC-labeled anti-mouse Ig compared to unstained cells detects the % of cells expressing surface Ig. More than 99% of ZK1 cells express F4/80 and CD11b antigens.

Phenotypic analysis of ZK cells revealed the presence of macrophage markers F4/80 and Mac-1 (CD11b), which are expressed only by mature macrophages, but not on polymorphonuclear leukocytes, lymphocytes, or fibroblasts [32,33]. Immunofluorescent labeling demonstrated that more than 99.5% of ZK1 cell population expressed F4/80 and CD11b (Fig. 4). Moreover, the surface expression of F4/80 and CD11b on ZK1 cells was greater than that seen on the AMJ2-C11 cell line (relative fluorescence of F4/80 and CD11b was 155.9 *vs *57.3 and 114.5 *vs *92.1 compared to AMJ2-C11, respectively). Similar results were observed in ZK2 and ZK6 cells (data not shown). In addition, all three clones ZK1, ZK2 and ZK6 adhered to plastic tissue culture surfaces, a feature characteristic of macrophages. However, similar to primary AMs isolated from MS^-/- ^mice, these ZK cells did not attach to the plastic as tightly as wild-type macrophages. These ZK cells and primary AMs deficient in MARCO and SR-AI/II receptors were readily detached by vigorously pipetting after cold PBS treatment for 5–10 min.

### Phagocytosis of opsonized SRBCs

**Figure 5:**
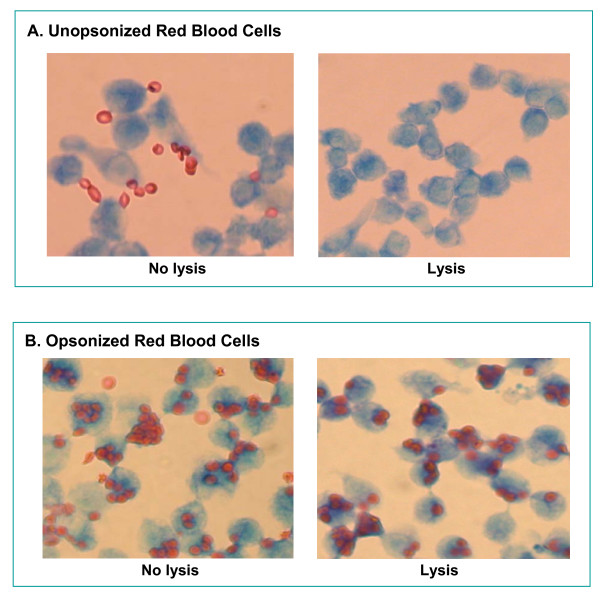
Fc-receptor on ZK1 cells mediated phagocytosis of opsonized sheep red blood cells (SRBC). ZK1 cells were plated at 1 × 10^6 ^cells/well in a 6-well plate containing a sterile micro cover glass per well in RPMI complete medium for overnight at 37°C. Unopsonized (as negative control) or preopsonized SRBC were plated on monolayer of ZK1 cells at a ratio of 20:1 and incubated at 37°C for 1 h. After removal of free SRBC by medium exchange and lysis by osmotic shock, the cells on the cover glass were fixed and stained with a modified Wright stain, subsequently examined by light microscopy. Panel **A**, ZK1 cells were unable to ingest unopsonized SRBC after lysis. Some free SRBC were present without lysis as background. Panel **B**, approximately 80% of ZK1 cells were positive phagocytosis of opsonized SRBC.

ZK1, ZK2 and ZK6 cell lines exhibited sheep erythrocyte receptor- and FcγR-mediated phagocytosis of opsonized sheep red blood cells (SRBCs) that are typical of macrophages [34,35]. In contrast to opsonized SRBCs, only rare unopsonized SRBCs appeared bound to ZK1 cells; most cells did not have any unopsonized SRBCs attached. After binding, these unopsonized SRBCs were easily lysed away (Fig. 5A). Approximately 80% of ZK1 cells were positive for FcγR-mediated phagocytosis of opsonized SRBCs (Fig. 5B). Similar results were seen in ZK2 and ZK6 clones (data not shown).

### Decreased binding and phagocytosis of unopsonized particles

**Figure 6:**
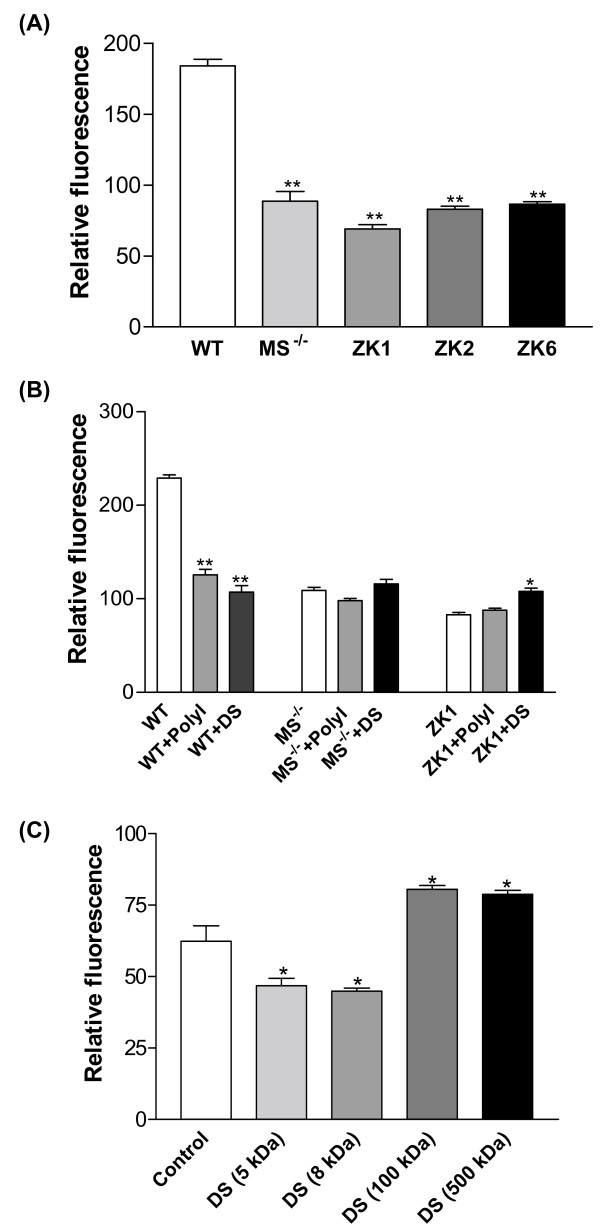
(**A**) ZK cell lines and primary AMs with MS^-/- ^significantly diminished phagocytosis of fluorescent latex beads. Wild type primary AMs were used as control. Values shown are the means ± SD from four separate experiments. **Significant difference from WT control, *P *< 0.001. (**B**) Polyinosinic acid (PolyI) had no effect but dextran sulfate (DS) increased binding by ZK1 cells. In contrast, PolyI and DS marked reduced wild-type AM binding of the latex beads. Data are expressed as the mean ± SD and compared to the control in each group (WT, MS^-/-^, ZK1, respectively). *Significant difference compared with ZK1 (*P *< 0.05); **Significant difference compared with WT (*P *< 0.001). PolyI and DS (500 kDa), 10 μg/ml each. (**C**) The inhibition of ZK1 cells binding of the latex beads by dextran sulfate was size-dependent. Only smaller 5-8-kDa dextran sulfate was able to inhibit ZK1 cells binding of the latex beads. Data were shown as means ± SD. * *P *< 0.05 versus control (n = 4). DS, 10 μg/ml.

To determine whether MS^-/- ^macrophages were able to bind and phagocytose unopsonized particles, uptake by ZK clones of unopsonized fluorescent latex beads was compared with that of primary AMs from MS^-/- ^mice and wild-type mice. Flow cytometry analysis revealed that all three ZK clones exhibited binding and phagocytic capacity of latex beads similar to primary AMs deficient in MARCO and SR-AI/II. All three clones showed significantly decreased uptake of fluorescent latex beads compared to the wild type primary AMs (Fig. 6A). Observed differences in phagocytic capacity between these clones and parental primary AMs from MS^-/- ^mice may reflect the heterogeneity seen in populations of primary alveolar macrophages.

In contrast to findings in AMs from wild-type mice, uptake of fluorescent latex beads by MS^-/- ^AMs and ZK cells is not inhibited by scavenger receptor ligands such as polyinosinic acid (PolyI) and dextran sulfate (DS, 500 kDa) at 10 μg/ml (Fig. 6B), nor by fucoidan (data not shown). Notably, dextran sulfate actually enhanced slightly the uptake of fluorescent latex beads by ZK1 cells (Fig. 6B). Furthermore, to determine whether the size of dextran sulfate molecules alters the effect on ZK1 cells' uptake of latex beads, we tested different sizes of DS in the binding/phagocytosis assay. The results indicated that only dextran sulfate with smaller molecular weight (5-8-kDa) inhibited binding, whereas larger 100-500-kDa dextran sulfate did not show inhibition of binding, but even enhanced binding (Fig. 6C).

**Figure 7:**
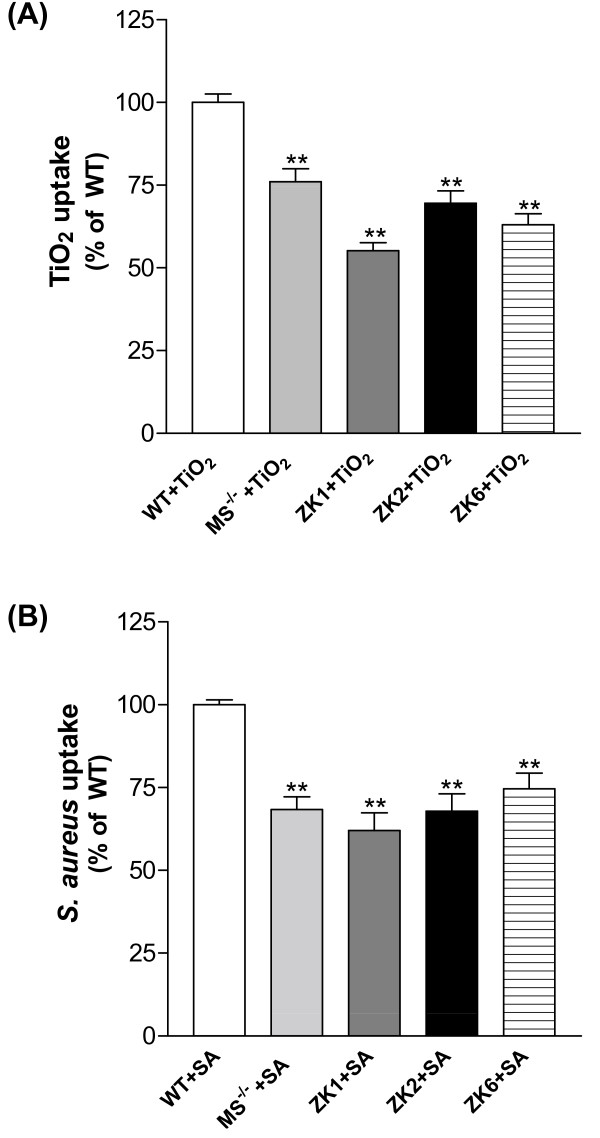
ZK cells and primary AMs with MS^-/- ^reduced binding and phagocytosis of TiO_2_ (**A**) and fluorescently labeled heat-killed *S. aureus *(**B**) in comparison to the wild-type primary AMs. TiO_2_, 50 μg/ml; the ratio of *S. aureus *to cells was 50:1. Values represent the mean ± SD. **Significant difference from WT control, *P *< 0.001, n = 4.

ZK cell lines also exhibited phagocytosis of titanium dioxide (TiO_2_) and fluorescently labeled heat-killed *Staphylococcus aureus*. However, similar to primary AMs from MS^-/- ^mice, all three ZK cell lines demonstrated marked reduced phagocytosis activity compared to primary AMs from their wild type mice (Fig. 7A and Fig. 7B). These results indicated that ZK cells persist in macrophage phagocytic capacity functionally, but like primary AMs deficient in MARCO and SR-AI/II, ZK cells were significantly impaired in phagocytosis compared to the WT AMs due to deficiency in scavenger receptors.

## Discussion

Alveolar macrophages play a central role in lung defense [3,36,37]. The class A scavenger receptors (SRA) MARCO and SR-AI/II are expressed on alveolar macrophages and function in innate defenses against inhaled pathogens and particles [7,8,10,11,17]. However, large number of murine alveolar macrophages with SRA deficient are rarely available for *in vitro *studies. To further investigate the role of MARCO and SR-AI/II receptors in uptake of unopsonized particles and bacteria, alveolar macrophages isolated by BAL from MARCO^-/- ^and SR-AI/II^-/- ^mice were immortalized and cloned for this study. Immortalization was conducted as described previously by infection of alveolar macrophages with J2 retrovirus [30]. In this present study, we described that three clones ZK1, ZK2 and ZK6 were obtained by limiting dilution and further characterized. Our PCR genotyping results verified these three clones, ZK1, ZK2 and ZK6 are MARCO^-/- ^and SR-AI/II^-/-^-deficient (Fig. 1). These cell lines are able to grow rapidly in RPMI or DMEM complete media in the absence of exogenous M-CSF or other growth factors, and their doubling time is 12–16 h on the average in RPMI complete medium (Fig. 2). Their viability after freezing in liquid nitrogen and recovery into culture is satisfactorily high. These cell lines are also stable during the culture over the past 24 months. Therefore, these cell lines provide the advantage over freshly isolated cells of an unlimited source of phenotypically and functionally homogeneous cell populations.

We showed here that all three of ZK cell lines responded in a manner comparable to that of primary murine alveolar macrophages. Morphologically, they are alveolar macrophage-like with Diff Quik, a modified Wright staining (Fig. 3). They all highly expressed macrophage antigens, F4/80 and CD11b on cell surfaces by immunofluorescent staining and flow cytometry assays (Fig. 4). ZK1, ZK2 and ZK6 clones exhibited macrophage FcγR-mediated phagocytosis of opsonized sheep red blood cells (Fig. 5A and Fig. 5B).

Functionally, all three ZK clones retain some reduced capacity for binding and phagocytosis of unopsonized particles (fluorescent latex beads and TiO_2_) and bacteria (*S. aureus*). Similar to primary alveolar macrophages from MARCO^-/- ^and SR-AI/II^-/- ^mice, they demonstrate marked decreased binding and phagocytosis of these particles compared to primary AMs from wild type mice (Fig. 6A and Fig. 7A and Fig. 7B). The results confirm that MARCO and SR-AI/II receptors on alveolar macrophages play critical roles in uptake of unopsonized environmental particles and bacteria. Moreover, the reduced, but still demonstrable, ability to bind unopsonized particles was not inhibited by scavenger ligands such as polyinosinic acid and dextran sulfate of 500 kDa (Fig. 6B) and fucoidan (data not shown). These results suggested that other additional, as yet unidentified, receptors can be involved in the binding and ingestion of unopsonized particles. We speculate that these putative receptor(s) are induced to compensate for the absence of scavenger receptors in cells from deficient animals. Our studies also found that only smaller dextran sulfate of 5-8-kDa inhibited the residual, non-scavenger receptor mediated binding of unopsonized fluorescent latex beads by ZK1 cells. However, larger dextran sulfate of 100-500-kDa did not inhibit and even enhanced uptake of fluorescent latex beads by ZK1 cells (Fig. 6C). Interestingly, Chicoine *et al *[38] have observed that dextran sulfate of 500 kDa but not smaller 5-8-kDa dextran sulfate has potent effects on calcium channel activity in brain tissue. Our previous studies found that a marked Ca^2+^-dependence of AM binding of fluorescent latex beads [7,36]. The potential interaction of alternate receptors with calcium signals in the uptake of unopsonized receptors is one avenue of investigation that can be addressed with these continuous cell lines.

## Methods and Materials

### Animals

SR-AI/II-deficient mice [6], and MARCO-deficient mice, constructed by Soininen and colleagues [39], both on the C57BL/6 background were maintained in Harvard School of Public Health animal facility under pathogen-free conditions. Both MARCO and SR-AI/II-deficient (MS^-/-^) mice were obtained by cross-breeding of the MARCO^-/- ^mice with SR-AI/II^-/- ^mice in our facility in accordance with NIH guide lines. Wild type C57BL/6 (WT) mice were purchased from Charles River Laboratories International, Inc (Wilmington, MA). Male mice between 9 to 14 weeks of age were used in the experiments. Animal experiments were conducted under a protocol #03827 approved by Harvard School of Public Health institutional animal use review committee.

### Reagents and particles

Titanium dioxide (TiO_2_) was obtained from Baker Chemicals (Phillipsburg, NJ). Latex beads (1.0 μm in diameter) that show green fluorescence after excitation at 488 nm were purchased from Interfacial Dynamics Co (Portland, OR). Fluorescently-labeled, heat-killed bacteria (*Staphylococcus aureus*) were obtained from Molecular Probes (Eugene, OR). All particles were suspended in balanced salts solution (BSS^- ^[124 mM NaCl, 5.8 mM KCl, 10 mM dextrose and 20 mM HEPES]) at stock solution and were probe sonicated for ~40 s (Model W-p200, setting 4; Ultrasonics, Plainview, NY) immediately prior to use. Dulbecco's modified Eagle's medium (DMEM) and fetal bovine serum (FBS) were obtained from American Type Culture Collection (ATCC, Manassas, VA); RPMI 1640 medium and PBS were purchased from BioWhittaker (Walkersville, MD); Gentamycin, penicillin/streptomycin, macrophage-colony stimulating factor (M-CSF), and Polybrene were obtained from Sigma (St. Louis, MO); MethoCult™ GF M3434, semi-solid medium was obtained from StemCell Technologies (Vancouver, Canada); Diff-Quik, a modified Wright-Giemsa stain was obtained from VWR (Boston, MA); Bovine washed red blood cells (RBCs) was purchased from Quad Five (Ryegate, MT); Rabbit IgG fraction of anti-sheep red blood cell was purchased from Rockland Inc (Gilbertsville, PA); Fluorescein isothiocyanate (FITC)-labeled anti-mouse F4/80 and FITC-labeled anti-mouse CD11b (Mac-1) were purchased from eBioscience Inc (San Diego, CA); FITC-labeled FcγIII/II receptor, and FITC-labeled rat isotype controls, IgG2a and IgG2b, and mAb 2.4G2 were obtained from BD BioScience Pharmingen (San Diego, CA). All chemical reagents not otherwise specified were obtained from Sigma-Aldrich.

### Cell line

AMJ2-C11 cell line was purchased from ATCC, and cultured in Dulbecco's Modified Eagle's Medium (DMEM) supplemented with 10% fetal bovine serum (FBS) and gentamycin (50 μg/ml). ZK cell lines in this study were cultured in RPMI 1640 complete medium, in which RPMI medium was supplemented with 10% FBS, L-glutamine (2 mM), penicillin (100 U/ml), streptomycin (100 μg/ml).

### Isolation of alveolar macrophages

Alveolar macrophages (AMs) were obtained by repeated brochoalveolar lavage with phosphate-buffered saline (PBS) from C57BL/6 mice or MARCO^-/- ^and SR-AI/II^-/- ^mice after being euthanized by intraperitoneal pentobarbital injection. Brochoalveolar lavage fluid (BAL) was immediately placed on ice, centrifuged at 200 × g for 10 min and the pellets were washed twice with cold PBS. Cell counts and viability were determined by using a hemocytometer and trypan blue dye exclusion. Cytocentrifuge preparations were stained with Diff-Quik, a modified Wright-Giemsa stain to allow differential analysis. All BAL samples contained greater than 95% AMs. AMs were adjusted to 1 × 10^6 ^cells/ml in DMEM conditioned medium (CM), which contained DMEM medium supplemented with 15% FBS, penicillin (100 U/ml), streptomycin (100 μg/ml), 10 mM HEPES, and 10 ng/ml of mouse macrophage colony stimulating factor (M-CSF).

### J2 recombinant retrovirus preparation

The AMJ2-C11 cell line was the source of the J2 retrovirus used [30]. The virus was prepared as described previously [30] with some modifications. Supernatants from exponentially growing cultures of AMJ2-C11 were collected and cell debris was removed by centrifugation (3000 g, rotor GH-3.8, Beckman) for 15 min. After clarification, the supernatants were filtered through a 0.45-μm membrane (Millipore, Bedford, MA), diluted 1/1 with DMEM conditioned Medium containing 6 μg/ml of polybrene (final concentration), and added to the primary cultures for overnight at 37°C.

### Immortalization of alveolar macrophages

Primary AMs were isolated by BAL and stimulated to proliferate in DMEM conditioned medium at a density of 1 × 10^6 ^cells/ml in a 6-well plate (Costar, Cambridge, MA), and incubated for 24 h at 37°C with 5% CO_2_. Nonadherent cells were washed off with warm medium 6–8 h later. After an additional incubation for 24 h in DMEM conditioned medium, the nonadherent cells were washed off again, resulting in a population of cells with predominantly macrophage (>99%) morphology as determined with the modified Wright's stain. Adherent AM monolayers were infected with J2 virus supernatants mixed with DMEM conditioned media and polybrene. After 24 h, excess virus was removed and cells were exposed to a second round of J2 infection under the same conditions to improve transfection efficiency. Clusters of rapidly proliferating cells were evident ca. 10 days after initial infection. After 1 month, cells were grown in regular DMEM medium without M-CSF. The virus transformed cells progressively increased in number. Several AM cell lines rapidly growing in semi-solid medium (0.96% methylcellulose) were isolated and subsequently recloned by limiting dilution method. Three clones designated as ZK1, ZK2, ZK6 were characterized using the assays described below.

### Cytochemical staining

ZK cells (5 × 10^4 ^cells/slide) were cytocentrifuged onto glass slides, air-dried and stained with Diff-Quik, a modified Wright-Giemsa stain. ZK cells were identified as alveolar macrophages by their large, dark nuclei and abundant pale, granular cytoplasm often containing numerous vacuoles. Primary AMs were isolated from the wild type mice and MS^-/- ^mice as controls.

### PCR genotyping of ZK cell lines

Total genomic DNA was isolated from cultured ZK cells as well as from wild type (WT) mouse tails according to the protocols described in the DNeasy Tissue Kit from QIAGEN (Valencia, CA). Primers used for SR-AI/II were: forward primer (for both WT and SR-AI/II^-/- ^mice), 5'-CAAGTGATA CATCTCAAGGTC-3'; reverse primer for WT, 5'-CTGTAGATTCACGGACTCTG-3', which amplify a 325-bp product, and reverse primer for SR-AI/II^-/-^, 5'GAGGAGTAGAAG GTGGCGCGAA-3', which amplify a 434-bp product. Primers used for MARCO WT allele are 5'-CAGCTGGGTCCATACCAGC-3' (forward primer) and 5'-CTGGAGAGCCTCGTTGACC-3' (reverse primer), which amplify a ca. 500-bp product; Primers used for MARCO mutant allele were 5'-CCACGCTCATCG ATAATTTCAC-3' (forward primer) and 5'-GCCTGCAGTGGCCGTCGTTTTA-3'(reverse primer), which amplify a ca. 850-bp product. The PCR was conducted on a MJ Research PTC-200 (Watertown, MA) under the following conditions (4 min at 94°C, followed by 30 cycles of 1 min at 94°C, 1 min at 58°C, and 1 min at 72°C, followed by 5 min at 72°C). PCR products (10–15 μl/lane) were resolved in a 2.0% agarose gel.

### Mean generation time

ZK-1, ZK-2 and ZK-6 cells were seeded in six-well plates at 2 × 10^4 ^cells/ml with RPMI complete medium and incubated at 37°C in 5% CO_2_-humidified air. At various time intervals (14 h and 18 h incubation), three wells of each cell line were detached by repeatedly pipetting after treatment with cold PBS for 10 min, and the cells from each well were counted with a hemacytometer. Cell viability was determined by trypan blue dye exclusion. Doubling time (mean generation time = mgt) was calculated according the formula: N = N_0_2^T/mgt^. N is the number of cells at any time T; N_0_, is the number of cells at an initial point.

### Detection of macrophage-associated cell-surface antigens by flow cytometry assay

Expression of CD11b (Mac-1) and F4/80 antigens was demonstrated by immunofluorescence staining of cell suspensions. To make a single cell suspension from cultured cell lines, cells were harvested by repeatedly pipetting with cold PBS. Cells were washed twice in a staining buffer (PBS containing 0.1% fetal bovine serum and sodium azide), and resuspended at 2 × 10^7^/ml. Fifty microlitters of each cell suspension were incubated with the following mAbs: pre-incubate the cells with 0.5 μg of mAb 2.4G2 (anti-mouse CD16/CD32, to block binding of FcγR) per million cells for 5–10 minutes on ice prior to staining, then add 0.25 μg/ml of FITC-labeled anti-mouse F4/80 or FITC-labeled anti-mouse CD11b to the cells in each tube, mix gently and incubate for 30 min in the dark. All staining procedures were carried out at 4°C. Cells were then washed 3 times and analyzed using a Coulter ELITE flow cytometer (Coulter Corporation, Miami, FL). At least 10,000 cells were collected for each histogram. Positive staining was determined by comparison with relevant FITC labeled rat IgG isotype.

### Phagocytosis of erythrocytes

Sheep red blood cells (SRBC) were opsonized by mixing fresh SRBC with appropriately titered rabbit anti-SRBC antiserum (Rockland, Gilbertsville, PA) for 30 min at room temperature, then the erythrocytes were washed, plated on monolayer of macrophages at a ratio of 20:1. Fc-receptor-dependent phagocytosis of IgG-coated bovine erythrocytes by ZK cell lines was performed as described by Mbawuike IN et al. [40] with some modification. Briefly, ZK cells were plated at 1 × 10^6 ^cells/well in a 6-well plate containing a sterile 22 × 22 mm micro cover glass (VWR Scientific) per well in RPMI complete medium. After adherence overnight at 37°C, the unattached cells were washed away by medium exchange, then replaced with 2 ml RPMI medium to each well containing ca. 2 × 10^7^/ml of SRBC and incubated at 37°C for 1 h. After removal of free SRBC by medium exchange and lysis by osmotic shock, the cells on the cover glass were fixed with 2% paraformaldehyde and stained with Diff-Quik stain. The percentage of ZK cells which phagocytosed SRBC was scored by light microscopy. Cells were deemed positive for phagocytosis if they ingested more than five SRBC. Cells uptake of unopsonized SRBC and cells without lysis were used as controls.

### Binding/phagocytosis of unopsonized particles using flow cytometry

Alveolar macrophage and ZK cell lines were collected and resuspended at 2 × 10^6 ^cells/ml in a balanced salt solution (BSS^+^) containing NaCl (124 mM), KCl (5.8 mM), dextrose (10 mM) and HEPES (20 mM), adding CaCl_2 _(0.3 mM) and MgCl_2 _(1.0 mM). In a 96-well ultra low adherent plate (Corning Inc., Corning, NY): fifty microliters of macrophages (100K cells) were mixed with 50 μl of TiO_2 _(final conc. 50 μg/ml) or fluorescent latex beads/bacteria (ratio of particles to cells: 50:1) and incubated at 37°C for 40 min. Cells and particles were mixed once more during the assay at 20 min. In some assays, MØs were preincubated with scavenger receptor inhibitors, polyinosinic acid (Poly-I, 10 μg/ml) and dextran sulfate (DS, 10 μg/ml) for 10 min at room temperature. After incubation, the plate was put on the ice and was added 200 μl of BSS^+ ^buffer to each well, then transferred to flow tubes for flow cytometry. Propidium iodide (PI) was added to each tube to separate live cells from dead cells. MØ uptake of particles was measured using the increase in the mean right angle scatter (RAS) caused by these granular materials. All cell types tested showed almost identical baseline RAS values (no differences between the macrophage lines), which increased after particle uptake. Fluorescent latex beads or bacteria binding is expressed as relative fluorescence.

### Statistical analysis

Experiments were performed, at minimum, in triplicate and all results represent the mean ± SD. We determined statistical significance between groups using the Student *t *test. For three or more groups, differences among groups were evaluated using One-way ANOVA on each group and followed by Dunnett's Multiple Comparison Test. A value of *P *< 0.05 was considered significant difference. All statistics and graphs were performed using Prism software, version 3.0 (GraphPad, San Diego, CA).

## List of abbreviations

**AM**: alveolar macrophage; **MØ**, macrophage; **SR**: scavenger receptor; **MARCO**: macrophage receptor with collagenous structure; **MS**^-/-^: MARCO and SRA-I/II-deficient; **WT**: wild type; **BAL**: brochoalveolar lavage; **CM**: conditioned medium; **M-CSF**: macrophage colony-stimulating factor; **DMEM**: Dulbecco's modified Eagle's medium; **Poly-I**: polyinosinic acid; **DS**: dextran sulfate; **TiO**_2_: titanium dioxide.

## Competing interests

The authors declare that they have no competing interests.

## Authors' contributions

**HZ **and **LK **conceived and designed the study. **HZ **worked on acquisition and interpretation of the data, and on drafting the manuscript.**AI **was involved in the binding/phagocytosis assay by flow cytometry and interpretation of the results. **LK **was the project leader; he has been involved in the analysis and interpretation of data, and in revising the manuscript critically for important intellectual content. All authors read and approved the final manuscript.

## References

[B1] Hocking WG, Golde DW (1979). The pulmonary-alveolar macrophage (second of two parts). N Engl J Med.

[B2] Hocking WG, Golde DW (1979). The pulmonary-alveolar macrophage (first of two parts). N Engl J Med.

[B3] Lohmann-Matthes ML, Steinmuller C, Franke-Ullmann G (1994). Pulmonary macrophages. Eur Respir J.

[B4] Twigg HL (2004). Macrophages in innate and acquired immunity. Semin Respir Crit Care Med.

[B5] Rivera R, Hutchens M, Luker KE, Sonstein J, Curtis JL, Luker GD (2007). Murine alveolar macrophages limit replication of vaccinia virus. Virology.

[B6] Suzuki H, Kurihara Y, Takeya M, Kamada N, Kataoka M, Jishage K, Ueda O, Sakaguchi H, Higashi T, Suzuki T (1997). A role for macrophage scavenger receptors in atherosclerosis and susceptibility to infection. Nature.

[B7] Kobzik L (1995). Lung macrophage uptake of unopsonized environmental particulates. Role of scavenger-type receptors. J Immunol.

[B8] Pearson AM (1996). Scavenger receptors in innate immunity. Curr Opin Immunol.

[B9] Palecanda A, Paulauskis J, Al-Mutairi E, Imrich A, Qin G, Suzuki H, Kodama T, Tryggvason K, Koziel H, Kobzik L (1999). Role of the scavenger receptor MARCO in alveolar macrophage binding of unopsonized environmental particles. J Exp Med.

[B10] Palecanda A, Kobzik L (2001). Receptors for unopsonized particles: the role of alveolar macrophage scavenger receptors. Curr Mol Med.

[B11] Arredouani MS, Palecanda A, Koziel H, Huang YC, Imrich A, Sulahian TH, Ning YY, Yang Z, Pikkarainen T, Sankala M (2005). MARCO is the major binding receptor for unopsonized particles and bacteria on human alveolar macrophages. J Immunol.

[B12] Kodama T, Freeman M, Rohrer L, Zabrecky J, Matsudaira P, Krieger M (1990). Type I macrophage scavenger receptor contains alpha-helical and collagen-like coiled coils. Nature.

[B13] Platt N, Haworth R, Darley L, Gordon S (2002). The many roles of the class A macrophage scavenger receptor. Int Rev Cytol.

[B14] Nakamura K, Funakoshi H, Tokunaga F, Nakamura T (2001). Molecular cloning of a mouse scavenger receptor with C-type lectin (SRCL)(1), a novel member of the scavenger receptor family. Biochim Biophys Acta.

[B15] Krieger M, Stern DM (2001). Series introduction: multiligand receptors and human disease. J Clin Invest.

[B16] Platt N, Gordon S (2001). Is the class A macrophage scavenger receptor (SR-A) multifunctional? – The mouse's tale. J Clin Invest.

[B17] Peiser L, Mukhopadhyay S, Gordon S (2002). Scavenger receptors in innate immunity. Curr Opin Immunol.

[B18] Hamilton RF, Thakur SA, Mayfair JK, Holian A (2006). MARCO mediates silica uptake and toxicity in alveolar macrophages from C57BL/6 mice. J Biol Chem.

[B19] Arredouani MS, Yang Z, Imrich A, Ning Y, Qin G, Kobzik L (2006). The Macrophage Scavenger Receptor SR-AI/II and Lung Defense against Pneumococci and Particles. Am J Respir Cell Mol Biol.

[B20] Taniyama T, Holden HT (1979). Direct augmentation of cytolytic activity of tumor-derived macrophages and macrophage cell lines by muramyl dipeptide. Cell Immunol.

[B21] Walker E, Warner NL, Chesnut R, Kappler J, Marrack P (1982). Antigen-specific. I region-restricted interactions in vitro between tumor cell lines and T cell hybridomas. J Immunol.

[B22] Uchida T, Ju S, Fay A, Liu Y, Dorf ME (1985). Functional analysis of macrophage hybridomas. I. Production and initial characterization. J Immunol.

[B23] Liu YN, Uchida T, Ju ST, Dorf ME (1985). Functional analysis of macrophage hybridomas. II. Antibody-independent tumor cytotoxicity and its dissociation from IL-1 production and Ia expression. Cell Immunol.

[B24] Blasi E, Mathieson BJ, Varesio L, Cleveland JL, Borchert PA, Rapp UR (1985). Selective immortalization of murine macrophages from fresh bone marrow by a raf/myc recombinant murine retrovirus. Nature.

[B25] Blasi E, Radzioch D, Merletti L, Varesio L (1989). Generation of macrophage cell line from fresh bone marrow cells with a myc/raf recombinant retrovirus. Cancer Biochem Biophys.

[B26] Roberson SM, Walker WS (1988). Immortalization of cloned mouse splenic macrophages with a retrovirus containing the v-raf/mil and v-myc oncogenes. Cell Immunol.

[B27] Darnbrough C, Slater S, Vass M, MacDonald C (1992). Immortalization of murine primary spleen cells by v-myc, v-ras, and v-raf. Exp Cell Res.

[B28] Cox GW, Mathieson BJ, Gandino L, Blasi E, Radzioch D, Varesio L (1989). Heterogeneity of hematopoietic cells immortalized by v-myc/v-raf recombinant retrovirus infection of bone marrow or fetal liver. J Natl Cancer Inst.

[B29] Birchenall-Roberts MC, Engelmann GL, Keller JR, Lohrey N, Ruscetti FW (1989). Retroviral v-myc infection of primary fetal liver cells: transformation of monocytes in vitro. Oncogene.

[B30] Palleroni AV, Varesio L, Wright RB, Brunda MJ (1991). Tumoricidal alveolar macrophage and tumor infiltrating macrophage cell lines. Int J Cancer.

[B31] Jozefowski S, Arredouani M, Sulahian T, Kobzik L (2005). Disparate regulation and function of the class A scavenger receptors SR-AI/II and MARCO. J Immunol.

[B32] Austyn JM, Gordon S (1981). F4/80, a monoclonal antibody directed specifically against the mouse macrophage. Eur J Immunol.

[B33] Blasi E, Radzioch D, Durum SK, Varesio L (1987). A murine macrophage cell line, immortalized by v-raf and v-myc oncogenes, exhibits normal macrophage functions. Eur J Immunol.

[B34] Crocker PR, Kelm S, Dubois C, Martin B, McWilliam AS, Shotton DM, Paulson JC, Gordon S (1991). Purification and properties of sialoadhesin, a sialic acid-binding receptor of murine tissue macrophages. Embo J.

[B35] Indik ZK, Park JG, Hunter S, Schreiber AD (1995). Structure/function relationships of Fc gamma receptors in phagocytosis. Semin Immunol.

[B36] Brain JD (1986). Toxicological aspects of alterations of pulmonary macrophage function. Annu Rev Pharmacol Toxicol.

[B37] Lehnert BE (1992). Pulmonary and thoracic macrophage subpopulations and clearance of particles from the lung. Environ Health Perspect.

[B38] Chicoine LM, Suppiramaniam V, Vaithianathan T, Gianutsos G, Bahr BA (2004). Sulfate- and size-dependent polysaccharide modulation of AMPA receptor properties. J Neurosci Res.

[B39] Arredouani M, Yang Z, Ning Y, Qin G, Soininen R, Tryggvason K, Kobzik L (2004). The scavenger receptor MARCO is required for lung defense against pneumococcal pneumonia and inhaled particles. J Exp Med.

[B40] Mbawuike IN, Herscowitz HB (1989). MH-S, a murine alveolar macrophage cell line: morphological, cytochemical, and functional characteristics. J Leukoc Biol.

